# Performance rating of the transmuted exponential distribution: an analytical approach

**DOI:** 10.1186/s40064-015-1590-6

**Published:** 2015-12-24

**Authors:** Enahoro A. Owoloko, Pelumi E. Oguntunde, Adebowale O. Adejumo

**Affiliations:** Department of Mathematics, Covenant University, Ota, Ogun State Nigeria; Department of Statistics, University of Ilorin, Ilorin, Kwara State Nigeria

**Keywords:** Estimation, Flexibility, Maximum likelihood estimation, Properties, Transmuted Exponential

## Abstract

In this article, the so called Transmuted Exponential (TE) distribution was applied to two real life datasets to assess its potential flexibility over some other generalized models. Various statistical properties of the TE distribution were also identified while the method of maximum likelihood estimation was used to estimate the model parameters.

## Background

Attempts to generalize the Exponential distribution have led to the developement of Beta Exponential distribution (Nadarajah and Kotz 2006), Kumaraswamy Exponential distribution (Cordeiro and de Castro 2011), Generalized Exponential distribution (Gupta and Kundu 1999, 2007) and Exponentiated Exponential distribution (Gupta 2001). These distributions have been found to be more flexibly than the Exponential distribution when applied to real life data sets.

Let X denotes a random variable, the probability density function (pdf) and the cumulative density function (cdf) of an Exponential distribution with parameter *θ* can be defined using an alternative parameterization as;

1 $$g\left( x \right) = \frac{1}{\theta }e^{{{-}\left( {\frac{x}{\theta }} \right)}} ;\quad\,x \ge ,\,\theta \text{ > }0$$

and

2 $$G\left( x \right) = 1{-}\frac{1}{\theta }e^{{{-}\left( {\frac{x}{\theta }} \right)}} ;\quad\,x \ge 0,\,\theta \text{ > }0$$

respectively.

where; *θ* is the scale parameter

Several generalized families of distributions have been proposed in the literature, for instance, the β-G; (Eugene et al. 2002), Kumaraswamy-G; (Cordeiro and de Castro 2011), Transmuted family of distributions; (Shaw and Buckley 2007), Gamma-G (type 1); (Zografos and Balakrishnan 2009), McDonald-G; (Alexander et al. 2012), Gamma-G (type 2); (Ristic et al. 2012), Gamma-G (type 3); (Torabi and Montazari 2012), Log-gamma-G; Amini et al. (2012), Exponentiated T-X; Alzaghal et al. (2013), Exponentiated-G (EG); (Cordeiro et al. 2013), Logistic-G; Torabi and Montazari (2014), Gamma-X; (Alzaatreh et al. 2013), Logistic-X; (Tahir et al. 2015), Weibull-X; (Alzaatreh et al. 2013), Weibull-G; (Bourguignon et al. 2014) and Beta Marshall-Olkin family of distributions; (Alizadeh et al. 2015) and many others are available in the literature.

Of interest to us in this article is the Transmuted family of distribution which was obtained using the quadratic rank transmutation map. The transmuted family of distributions has been adopted by several notable authors to generalize known theoretical models, the Transmuted Weibull distribution; Aryal and Tsokos (2011), Transmuted Rayleigh distribution; (Merovci 2013), Transmuted Exponentiated Modified Weibull distribution; (Ashour and Eltehiwy 2013a), Transmuted Modified Weibull distribution; Khan and King (2013), Transmuted Lomax distribution; (Ashour and Eltehiwy 2013b), Transmuted Exponentiated Gamma distribution; Hussian (2014), Transmuted Inverse Rayleigh distribution; Ahmad et al. (2014), Transmuted Pareto distribution; (Merovci and Puka 2014), Transmuted Inverse Weibull distribution; (Khan et al. 2014), Transmuted Modified Inverse Weibull Distribution; (Elbatal 2013), Transmuted Additive Weibull distribution; (Elbatal and Aryal 2013), Transmuted Complementary Weibull Geometric Distribution; (Afify et al. 2014), Transmuted Inverse Exponential distribution; (Oguntunde and Adejumo 2015), Transmuted Size-Biased Exponential distribution; Ahmad et al. (2015) and Transmuted Gompertz distribution; (Abdul-Moniem and Seham 2015); are some known examples in the literature.

The aim of this article is to obtain the Transmuted Exponential (TE) distribution as a special case of Transmuted Weibull distribution following the content of Aryal and Tsokos (2011) and to assess its flexibility over some other generalized models using real life data sets.

The rest of this article is organized as follows; in "The Transmuted Exponential (TE) distribution: existing and more results", the TE distribution, its properties and various statistical properties are discussed, real life applications with respect to some other well-known generalized models shall be discussed in "Application", followed by concluding remark. The R-code for the analysis is provided as “Appendix”.

## The Transmuted Exponential (TE) distribution: existing and more results

A random variable X is said to have a transmuted distribution function if its pdf and cdf are respectively given by;

3 $$f\left( x \right) = g\left( x \right)\left[ {1 + \lambda - 2\lambda G\left( x \right)} \right]$$

4 $$F\left( x \right) = \left( {1 + \lambda } \right)G\left( x \right) - \lambda \left[ {G\left( x \right)} \right]^{2}$$

where; *x* > 0, and $\left| \lambda \right| \le 1$ is the transmuted parameter

*G*(*x*) is the cdf of the baseline distribution.

*f*(*x*) and *g*(*x*) are the associated pdf of *F*(*x*) and *G*(*x*), respectively.

When *λ* = 0; Eqs. (3) and (4) reduces to the baseline distribution.

If the parameter *η* = 1 in Eqs. (4) and (5) of Aryal and Tsokos (2011), we have the pdf and the cdf of the TE distribution as;

5 $$f\left( x \right) = \frac{1}{\theta }e^{{ - \left( {\frac{x}{\theta }} \right)}} \left[ {1 - \lambda + 2\lambda e^{{ - \left( {\frac{x}{\theta }} \right)}} } \right]$$

and;

6 $$F\left( x \right) = \left[ {1 - e^{{ - \left( {\frac{x}{\theta }} \right)}} } \right]\left[ {1 + \lambda e^{{ - \left( {\frac{x}{\theta }} \right)}} } \right]$$

Respectively.

For *x* > 0, *θ* > 0, $$\left| {\lambda \le 1} \right|$$

where;

*θ* is the scale parameter

*λ* is the transmuted parameter

### Special case

**Fig. 1:**
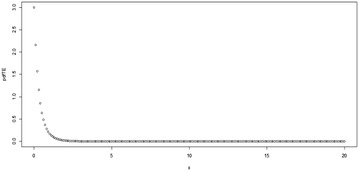
Plot for the pdf of TE distribution at (*θ* = 0.5, *λ* = 0.5)

**Fig. 2:**
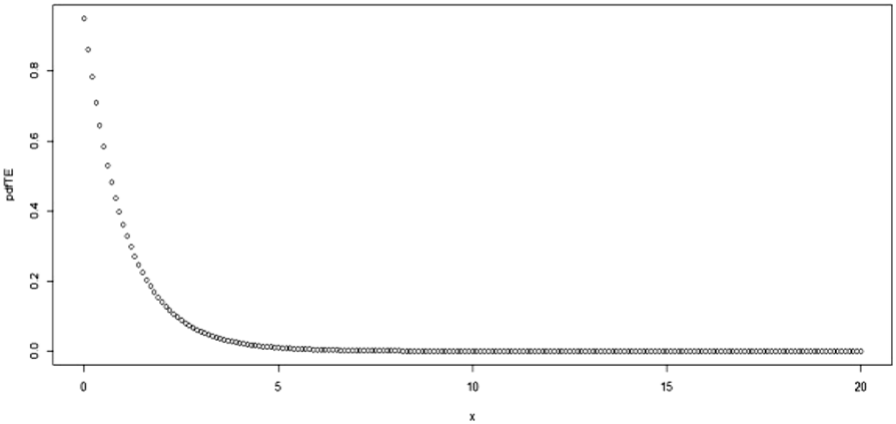
Plot for the pdf of TE distribution at (*θ* = 2, *λ* = 0.9)

**Fig. 3:**
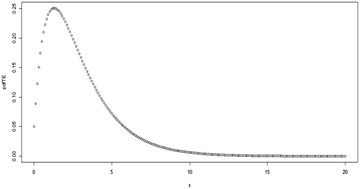
Plot for the pdf of TE distribution at (*θ* = 2, *λ* = −0.9)

**Fig. 4:**
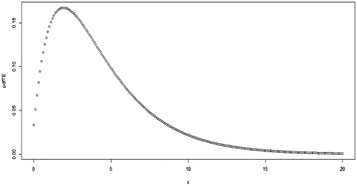
Plot for the pdf of TE distribution at (*θ* = 3, *λ* = −0.9)

**Fig. 5:**
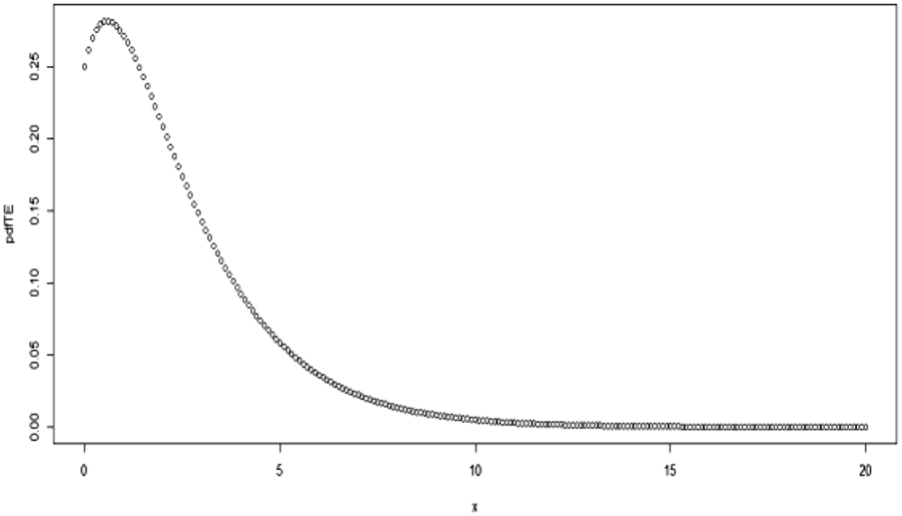
Plot for the pdf of TE distribution at (*θ* = 2, *λ* = − 0.5)

**Fig. 6:**
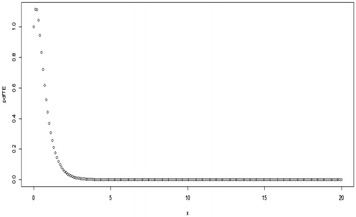
Plot for the pdf of TE distribution at (*θ* = 0.5, *λ* = − 0.5)

For *λ* = 0, Eq. (5) reduces to give the pdf of the Exponential distribution. Some possible plots for the pdf of the TE distribution at some selected parameter values are shown in Figs. 1, 2, 3,4, 5 and 6;

Depending on the parameter values, it can be observed from the figures above that the shape of the TE distribution could be decreasing, or inverted bathtub (unimodal). It should also be noted that $\left| \lambda \right| \le 1$.

### Moments of the Transmuted Exponential distribution

Let X denote a continuous random variable, the rth moment is given by;

$$E\left( {X^{r} } \right) = \int\limits_{ - \infty }^{\infty } {x^{r} } f\left( x \right)dx$$

Therefore, the rth moment of the TE distribution can be derived from;

7 $$E\left( {X^{r} } \right) = \int\limits_{ - \infty }^{\infty } {x^{r} } \frac{1}{\theta }e^{{ - \left( {\frac{x}{\theta }} \right)}} \left[ {1 - \lambda + 2\lambda e^{{ - \left( {\frac{x}{\theta }} \right)}} } \right]dx$$

This can be obtained directly from Eq. (6) of 8 when *η* = 1 as;

8 $$E\left( {X^{r} } \right) = \theta^{r} \varGamma \left( {1 + r} \right)\left\{ {1 - \lambda + \lambda 2^{ - r} } \right\}$$

This can further be expressed as;

9 $$E\left( {X^{r} } \right) = \theta^{r} r!\left\{ {1 - \lambda + \lambda 2^{ - r} } \right\}$$

It is obvious that for *r* = 1;

10 $$E\left( X \right) = \theta \left( {\frac{2 - \lambda }{2}} \right)$$

Other higher order moments can be derived at *r* > 1 from Eq. (9). The table of values (at selected values) for the mean of TE distribution is provided in Table 1

**Table 1:** Table of means for the Transmuted Exponential distribution

	*λ* = −0.1	*λ* = −0.4	*λ* = −0.7	*λ* = −1.0	*λ* = 0	*λ* = 0.1	*λ* = 0.4	*λ* = 0.7	*λ* = 1.0
*θ* = 1	1.05	1.20	1.35	1.50	1.00	0.95	0.80	0.65	0.50
*θ* = 2	2.10	2.40	2.70	3.00	2.00	1.90	1.60	1.30	1.00
*θ* = 3	3.15	3.60	4.05	4.50	3.00	2.85	2.40	1.95	1.50
*θ* = 4	4.20	4.80	5.40	6.00	4.00	3.80	3.20	2.60	2.00
*θ* = 5	5.25	6.00	6.75	7.50	5.00	4.75	4.00	3.25	2.50
*θ* = 6	6.30	7.20	8.10	9.00	6.00	5.70	4.80	3.90	3.00
*θ* = 7	7.35	8.40	9.45	10.50	7.00	6.65	5.60	4.55	3.50
*θ* = 8	8.40	9.60	10.80	12.00	8.00	7.60	6.40	5.20	4.00
*θ* = 9	9.45	10.80	12.15	13.50	9.00	8.55	7.20	5.85	4.50
*θ* = 10	10.50	12.00	13.50	15.00	10.00	9.50	8.00	6.50	5.00

.

### Quantile function and median of the Transmuted Exponential distribution

The quantile function *x*_*q*_ of the TE distribution can be obtained as the inverse of Eq. (6) and in particular, when *η* = 1 in Eq. (7) of (Aryal and Tsokos (2011)) as;

11 $$x_{q} = \theta \left[ { - \ln \left\{ {1 - \left( {\frac{{1 + \lambda - \sqrt {\left( {1 + \lambda } \right)^{2} - 4\lambda q} }}{2\lambda }} \right)} \right\}} \right]$$

The median of the TE distribution can be obtained from Eq. (11) at *q* = 0.5 as;

12 $$x_{0.5} = \theta \left[ { - \ln \left( {\frac{{\lambda - 1 + \sqrt {1 + \lambda^{2} } }}{2\lambda }} \right)} \right]$$

The lower quartile and upper quartile can also be derived from Eq. (11) when *q* = 0.25 and *q* = 0.75 respectively.

Random numbers from the TE distribution can be generated using the method of inversion;

13 $$X = \theta \left[ { - \ln \left\{ {1 - \left( {\frac{{1 + \lambda - \sqrt {\left( {1 + \lambda } \right)^{2} - 4\lambda u} }}{2\lambda }} \right)} \right\}} \right]$$

where; *u* ∼ *U*(0, 1).

### Reliability analysis of the Transmuted Exponential distribution

Mathematically, the survival function is given by;

14 $$S\left( x \right) = 1 - F\left( x \right)$$

Therefore, the survival function for the TE distribution can be simplified to give;

15 $$S\left( x \right) = \lambda e^{{ - 2\left( {\frac{x}{\theta }} \right)}} - \left( {\lambda - 1} \right)e^{{ - \left( {\frac{x}{\theta }} \right)}}$$

The hazard function is mathematically given by;

16 $$h\left( x \right) = \frac{f\left( x \right)}{1 - F\left( x \right)}$$

Therefore, the expression for the hazard function (or failure rate) of the TE distribution is given by;

17 $$h\left( x \right) = \frac{{\frac{1}{\theta }\left[ {1 - \lambda + 2\lambda e^{{ - \left( {\frac{x}{\theta }} \right)}} } \right]}}{{\left[ {\lambda e^{{ - \left( {\frac{x}{\theta }} \right)}} + 1 - \lambda } \right]}}$$

**Fig. 7:**
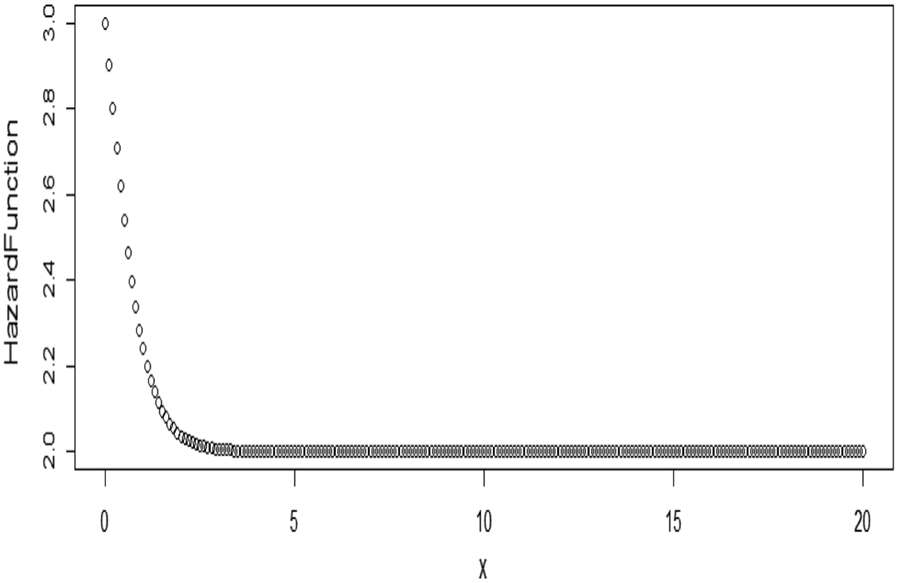
Plot for the hazard function of TE distribution at (*θ* = 0.5, *λ* = 0.5)

**Fig. 8:**
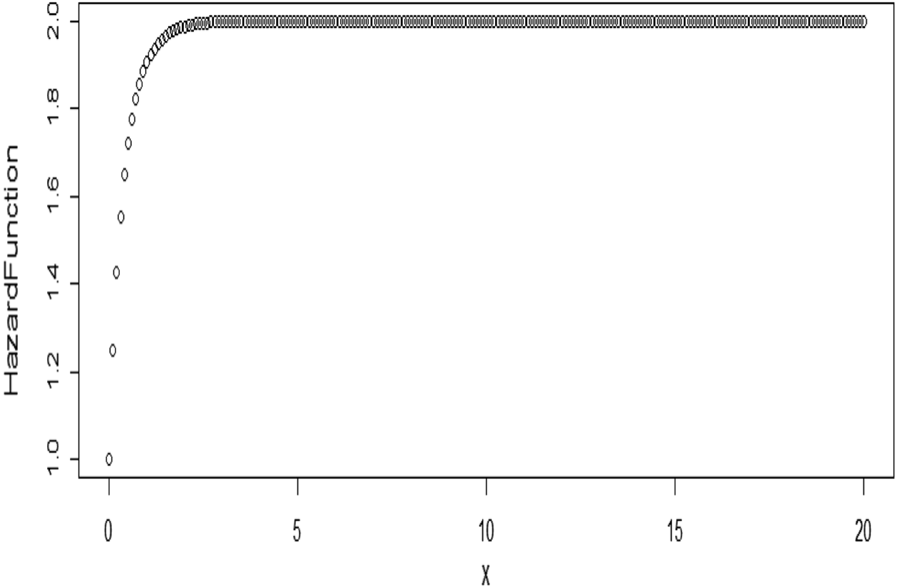
Plot for the hazard function of TE distribution at (*θ* = 0.5, *λ* = − 0.5)

**Fig. 9:**
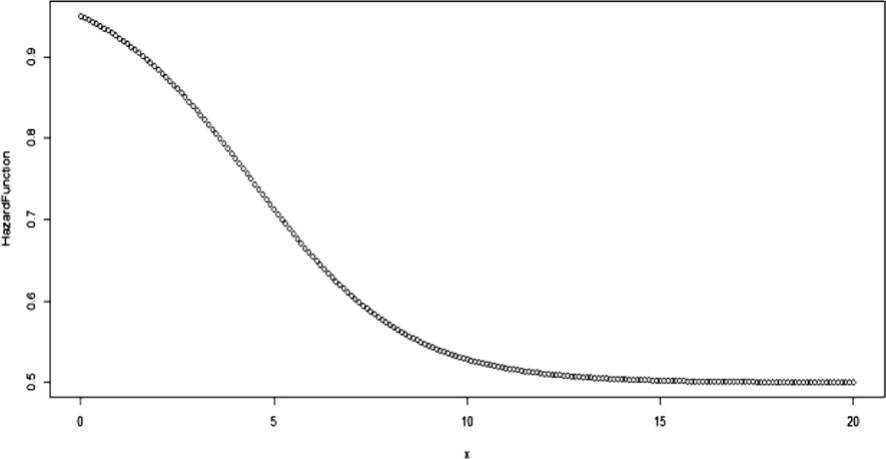
Plot for the hazard function of TE distribution at (*θ* = 2, *λ* = 0.9)

**Fig. 10:**
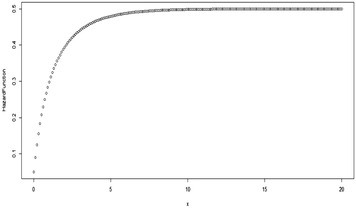
Plot for the hazard function of TE distribution at (*θ* = 2, *λ* = −0.9)

Some possible plots for the failure rate of the TE distribution at some selected parameter values are shown in Figs. 7, 8, 9 and 10;

### Parameter estimation and inference for the Transmuted Exponential distribution

We make use of the method of maximum likelihood estimation (MLE) to estimate the parameters of the TE distribution. Let *X*_1_, *X*_2_, …, *X*_*n*_ be a sample of size ‘n’ from the TE distribution, the likelihood function is given by;

$$L\left( {X_{1} ,X_{2} , \ldots ,X_{n} |\theta ,\lambda } \right) = \left( {\frac{1}{\theta }} \right)^{n} e^{{{{-}}\sum\limits_{{i{\text{-}}1}}^{n} {\left( {\frac{{x_{i} }}{\theta }} \right)} }} \prod\limits_{{i = 1}}^{n} {\left[ {1{{-}}\lambda {\text{ + }}2\lambda e^{{{{-}}\left( {\frac{{x_{i} }}{\theta }} \right)}} } \right]}$$

Let *l* = log *L*;

$$l = n\log \left( {\frac{1}{\theta }} \right) - \sum\limits_{i = 1}^{n} {\left( {\frac{{x_{i} }}{\theta }} \right)} + \sum\limits_{i = 1}^{n} {\log \left[ {1 - \lambda + 2\lambda e^{{ - \left( {\frac{{x_{i} }}{\theta }} \right)}} } \right]}$$

Therefore;

$$l = - n\log \theta - \sum\limits_{i = 1}^{n} {\left( {\frac{{x_{i} }}{\theta }} \right)} + \sum\limits_{i = 1}^{n} {\log \left[ {1 - \lambda + 2\lambda e^{{ - \left( {\frac{{x_{i} }}{\theta }} \right)}} } \right]}$$

Differentiating *l* with respect to *θ* and *λ* respectively gives;

18 $$\frac{\partial l}{\partial \theta } = - \frac{n}{\theta }\sum\limits_{i = 1}^{n} {\left[ {1 - \left( {\frac{{x_{i} }}{\theta }} \right)} \right]} + \frac{2\lambda }{\theta }\sum\limits_{i = 1}^{n} {\frac{{\left( {\frac{{x_{i} }}{\theta }} \right)e^{{ - \left( {\frac{{x_{i} }}{\theta }} \right)}} }}{{\left[ {1 - \lambda + 2\lambda e^{{ - \left( {\frac{{x_{i} }}{\theta }} \right)}} } \right]}}}$$

19 $$\frac{\partial l}{\partial \lambda } = \sum\limits_{i = 1}^{n} {\frac{{2e^{{ - \left( {\frac{{x_{i} }}{\theta }} \right)}} - 1}}{{\left[ {1 - \lambda + 2\lambda e^{{ - \left( {\frac{{x_{i} }}{\theta }} \right)}} } \right]}}}$$

Equating Eqs. (18) and (19) to zero and solving the resulting nonlinear system of equations gives the maximum likelihood estimates of parameters *θ* and *λ*.

We obtain the 2 × 2 observed information matrix through;

$$\left( {\begin{array}{*{20}l} {\hat{\theta }} \\ {\hat{\lambda }} \\ \end{array} } \right)\sim N\left[ {\left( {\begin{array}{*{20}c} \theta \\ \lambda \\ \end{array} } \right),\left( {\begin{array}{*{20}c} {\hat{V}_{{11}} } & {\hat{V}_{{12}} } \\ {\hat{V}_{{21}} } & {\hat{V}_{{22}} } \\ \end{array} } \right)} \right]$$

where;

20 $$V^{ - 1} = - E\left[ {\begin{array}{*{20}c} {\frac{{\partial^{2} l}}{{\partial \theta^{2} }}} & {\frac{{\partial^{2} l}}{\partial \theta \partial \lambda }} \\ {\frac{{\partial^{2} l}}{\partial \theta \partial \lambda }} & {\frac{{\partial^{2} l}}{{\partial \lambda^{2} }}} \\ \end{array} } \right]$$

The solution of the inverse matrix of the observed information matrix in Eq. (20) gives the asymptotic variance and co-variance of the maximum likelihood estimators $\hat{\theta }$ and $\hat{\lambda }$. The approximate $100 \,(1\;{ - }\;\alpha ) \;\%$ asymptotic confidence interval (CI) for *θ* and *λ* are given by;

21 $$\hat{\theta }\; \pm \;Z_{{\alpha /2}} \sqrt {\hat{V}_{{11}} } ;\quad \hat{\lambda } \pm \;Z_{{\alpha /2}} \sqrt {\hat{V}_{{22}} }$$

where; $Z_{\alpha/2}$ is the *α*-th percentile of the standard normal distribution.

## Application

The models to be compared in this section include the TE distribution, Beta Exponential distribution, Generalized Exponential Distribution and the Exponentiated Exponential distribution. The analyses were performed with the aid of R software.

*Data Set I*. The first data represents the life of fatigue fracture of Kevlar 373/epoxy subjected to constant pressure at 90 % stress level until all had failed. The data was extracted from (Abdul-Moniem and Seham 2015) and it has previously been used by Barlow et al. (1984). The data is as follows;

0.0251, 0.0886, 0.0891, 0.2501, 0.3113, 0.3451, 0.4763, 0.5650, 0.5671, 0.6566, 0.6748, 0.6751, 0.6753, 0.7696, 0.8375, 0.8391, 0.8425, 0.8645, 0.8851, 0.9113, 0.9120, 0.9836, 1.0483, 1.0596, 1.0773, 1.1733, 1.2570, 1.2766, 1.2985, 1.3211, 1.3503, 1.3551, 1.4595, 1.4880, 1.5728, 1.5733, 1.7083, 1.7263, 1.7460, 1.7630, 1.7746, 1.8275, 1.8375, 1.8503, 1.8808, 1.8878, 1.8881, 1.9316, 1.9558, 2.0048, 2.0408, 2.0903, 2.1093, 2.1330, 2.2100, 2.2460, 2.2878, 2.3203, 2.3470, 2.3513, 2.4951, 2.5260, 2.9911, 3.0256, 3.2678, 3.4045, 3.4846, 3.7433, 3.7455, 3.9143, 4.8073, 5.4005, 5.4435, 5.5295, 6.5541, 9.0960.

The summary of the data is provided in Table 2;

**Table 2:** Summary of data on fatigue fracture of Kevlar 373/epoxy at 90 % stress level (to four decimal places)

Min.	Q_1_	Q_2_	Q_3_	Mean	Max.	Variance	Skewness	Kurtosis
0.0251	0.9048	1.7360	2.2960	1.9590	9.0960	2.4774	1.9406	8.1608

The performance of the Transmuted Exponential distribution with respect to the Beta Exponential, Generalized Exponential and Exponentiated Exponential distributions using the data on fatigue fracture is given in Table 3.

**Table 3:** Performance rating of selected models

Distributions	Estimates	Log-likelihood	AIC
Transmuted Exponential (*θ*, *λ*)	*θ* = 1.3763, *λ* = −0.8487	−121.5166	247.0331
Beta Exponential (*a*, *b*, *θ*)	*a* = 1.6797, *b* = 1.5085, *θ* = 0.4849	−122.2275	250.4551
Generalized Exponential (*a*, *θ*)	*a* = 1.70949, *θ* = 0.70279	−122.2436	248.4872
Exponentiated Exponential (*a*, *θ*)	*a* = 39.969318, *θ* = 0.012770	−127.1143	258.2287

*Data Set II.* The second data set represents the monthly actual taxes revenue (in 1000 million Egyptian pounds) in Egypt between January 2006 and November 2010. The data was extracted from Nassar and Nada (2011). The data is as follows;

5.9, 20.4, 14.9, 16.2, 17.2, 7.8, 6.1, 9.2, 10.2, 9.6, 13.3, 8.5, 21.6, 18.5, 5.1, 6.7, 17, 8.6, 9.7, 39.2, 35.7, 15.7, 9.7, 10, 4.1, 36, 8.5, 8, 9.2, 26.2, 21.9, 16.7, 21.3, 35.4, 14.3, 8.5, 10.6, 19.1, 20.5, 7.1, 7.7, 18.1, 16.5, 11.9, 7, 8.6, 12.5, 10.3, 11.2, 6.1, 8.4, 11, 11.6, 11.9, 5.2, 6.8, 8.9, 7.1, 10.8.

The summary of the data is provided in Table 4.

**Table 4:** Summary of data on tax revenue (to two decimal places)

Min.	Q_1_	Q_2_	Q_3_	Mean	Max.	Variance	Skewness	Kurtosis
4.10	8.45	10.60	16.85	13.49	39.20	64.83	1.57	5.26

The performance of the Transmuted Exponential distribution with respect to the Beta Exponential distribution, Generalized Exponential distribution and the Exponentiated Exponential distribution is as shown in Table 5.

**Table 5:** Performance rating of selected models

Distributions	Estimates	Log-likelihood	AIC
Transmuted Exponential (*θ*, *λ*)	*θ* = 3.862 × 10^5^, *λ* = 9.389 × 10^−4^	−83.44494	170.8899
Beta Exponential (*a*, *b*, *θ*)	*a* = 63.52239, *b* = 0.16957, *θ* = 0.76882	−187.9398	381.8795
Generalized Exponential (*a*, *θ*)	*a* = 5.53040, *θ* = 0.17867	−191.2235	386.4471
Exponentiated Exponential (*a*, *θ*)	*a* = 11.755728, *θ* = 0.006307	−212.5068	429.0136

## Discussion

The model corresponding to the lowest Akaike Information Criteria (AIC) or the highest Log-likelihood value is regarded as the ‘best’ model. In this case, the TE distribution has the lowest AIC value with 247.0331 and 170.8899 respectively. Also, it has the highest value of Log-likelihood of −121.5166 and −83.44494 respectively. Hence, it can be regarded as a better model for the data used.

## Conclusion

This article studies the performance of the TE distribution with respect to some other generalized models. The shape of the TE distribution could be decreasing or unimodal (depending on the value of the parameters). The TE distribution appeared to be better than the Beta Exponential distribution, Generalized Exponential distribution and the Exponentiated Exponential distribution in terms of flexibility when applied two real life data. The criteria used are the Log-likelihood value and the AIC.
